# Enhanced cancer immunotherapy via synergistic action of NO-Donor nanoparticles (Nano^ARG^) and PD-1 antibody

**DOI:** 10.1080/14686996.2025.2538430

**Published:** 2025-07-29

**Authors:** Ting Mei, Xin Zhang, Xiaolu Hou, Yukio Nagasaki

**Affiliations:** a School of Chinese Materia Medica, Beijing University of Chinese Medicine, Beijing, China; b Department of Materials Science, Faculty of Pure and Applied Sciences, University of Tsukuba, Tsukuba Ibaraki, Japan; c Master’s School in Medical Sciences, Graduate School of Comprehensive Human Sciences, University of Tsukuba, Tsukuba, Ibaraki, Japan; d Center for Research in Radiation and Earth System Sciences (CRiES), University of Tsukuba, Tsukuba, Ibaraki, Japan; e Department of Chemistry, Graduate School of Science, The University of Tokyo, Tokyo, Bunkyo-ku, Japan; f High-value Biomaterials Research and Commercialization Center (HBRCC), National Taipei University of Technology, Taipei, Taiwan; g Center for Applied Nanomedicine, National Cheng Kung University, Tainan, Taiwan

**Keywords:** Tumor-associated macrophages, nanoparticle-based nitric oxide donor, immune checkpoint inhibitor (PD-1 antibody), cancer immunotherapy, synergistic anti-tumor effect, poly(L-arginine)-based polymer micelles

## Abstract

This study explores the synergistic potential of PEG-b-P(L-Arg)-based polyion complex micelles (Nano^ARG^s) combined with an immune checkpoint inhibitor (PD-1 antibody) for cancer immunotherapy. Comprehensive experiments, including micelle preparation, *in vivo* anti-tumor activity evaluation, nitric oxide (NO) quantification, and immunofluorescence analysis, revealed significant insights. Nano^ARG^s exhibited a biphasic effect on tumor growth: high doses inhibited tumor growth through NO generated from liberated Arg, whereas low doses promoted tumor progression. The combination treatment demonstrated significant synergistic anti-tumor activity without notable adverse effects, and treated mice tolerated the regimen well. This approach elevated NO levels in serum and tumor tissues, enhanced immune cell infiltration into tumor tissues, and facilitated the polarization of tumor-associated macrophages to the M1 phenotype. PD-1 antibody further amplified these effects by blocking PD-1/PD-L1 interactions and reactivating T cells. These results underscore the therapeutic potential of this novel approach, providing a foundation for optimizing tumor immunotherapy strategies and advancing clinical applications. Future research will focus on elucidating the mechanisms of action and expanding the scope of this promising treatment.

## IMPACT STATEMENT

Cancer immunotherapy shows great promise, yet its full potential remains unexploited. This study pioneers a novel combination therapy using PEG-b-P(L-Arg)-based polyion complex micelles (Nano^ARG^s) with a PD-1 antibody, delivering an unprecedented synergistic anti-tumor effect. Different from traditional nitric oxide (NO) donors in cancer therapy that have trouble with imprecise release control, Nano^ARG^s are innovatively developed as cascade-type NO donors, achieving precise NO release control and a unique biphasic anti-tumor effect, which prior research has not explored. While individual therapies of Nano^ARG^s or PD-1 antibody have their limitations, this study is the first to prove their combined remarkable synergistic anti-tumor effect, with enhanced efficacy and minimal toxicity in mouse models, offering a new treatment option distinct from monotherapies. Moreover, this study is the first to deeply explore the immunomodulatory mechanisms, revealing unique ways of increasing NO levels, polarizing tumor-associated macrophages to the anti-tumor M1 phenotype, and reactivating T cells, providing new insights not previously described in the literature and laying a foundation for optimized cancer immunotherapies.

## GRAPHICAL ABSTRACT

**Figure:**
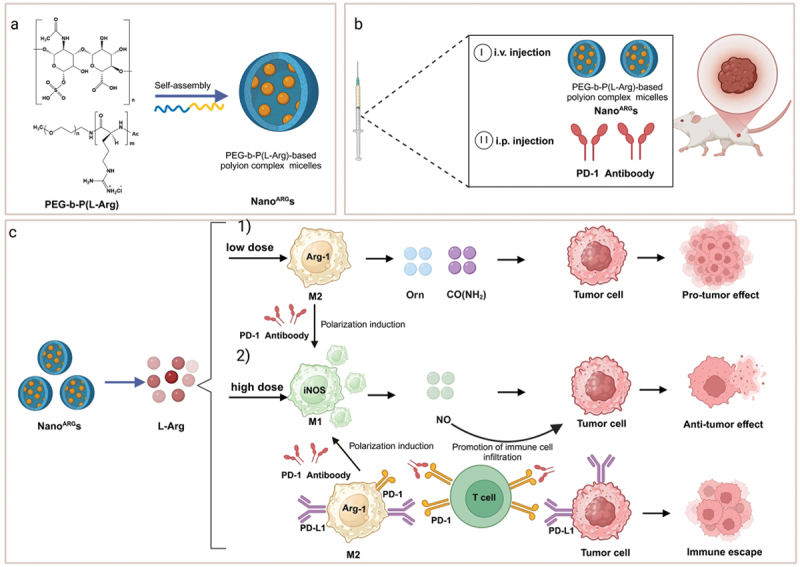

## 1. Introduction

Immunotherapy has emerged as a promising alternative to traditional cancer treatments such as surgery, chemotherapy, and radiation therapy [1]. Among various approaches, targeting the tumor microenvironment, particularly Tumor-Associated Macrophages (TAMs), has gained significant attention. TAMs exist in two phenotypes: M1 macrophages, which exhibit anti-tumor properties via pro-inflammatory cytokine secretion and tumor cell phagocytosis, and M2 macrophages, which promote tumor progression through immune-suppressive cytokines and angiogenesis [2].

The metabolic pathways of L-arginine (L-Arg) in TAMs highlight these contrasting roles: M1 macrophages produce nitric oxide (NO) via inducible nitric oxide synthase (iNOS), mediating cytotoxic effects [3], while M2 macrophages utilize L-Arg to generate polyamines via arginase-1-mediated metabolism, facilitating tumor growth and suppressing immune response [4]. The PD-1/PD-L1 axis also influences TAM phenotype and function, with PD-1 antibody (PD-1 Ab) therapy reprogramming TAMs from the M2 to the M1 phenotype, enhancing immune cell infiltration and T cell activation [5–7].

Recent advancements in NO delivery systems have employed diverse nanotechnological strategies for tumor-targeted, stimuli-responsive release. For instance, Sung et al. developed lipid-PLGA nanoparticles (NanoNO) that leverage the EPR effect for tumor accumulation, normalizing vasculature to enhance anti-tumor immunity and drug delivery efficiency [8]. Shi et al. reported a near-infrared (NIR)-triggered platform (EArgFe@Ce6), where L-arginine is decomposed to NO via ROS, enabling synergistic photodynamic, photothermal, and gas therapy [9]. Fan et al. introduced PEG-USMS-SNO nanoparticles that release NO upon X-ray exposure, sensitizing hypoxic tumors to radiotherapy [10], while Chung et al. demonstrated hollow microspheres generating NO bubbles under acidic conditions to reverse P-glycoprotein-mediated multidrug resistance [11]. These approaches highlight the versatility of NO-based nanomedicines in addressing tumor microenvironmental complexities through passive targeting, light/radiation/pH-responsive mechanisms. Building on these diverse NO delivery strategies, recent investigations have further explored the therapeutic potential of NO in combination with other immunotherapies, demonstrating the synergistic potential of NO donors with PD-1 Ab to enhance T cell infiltration and tumor regression [12]. However, challenges such as NO’s instability, toxicity and administration complexity limit its clinical application [13–16]. To address these challenges, we developed poly(ethylene glycol)-block-poly(L-arginine) (i.e. PEG-b-P(L-Arg)) block copolymer-based polyion complex (PIC) micelles (Nano^ARG^s), a cascade-type NO donor system. Nano^ARG^s are self-assembled from cationic PEG-b-P(L-Arg) and anionic chondroitin sulfate (CS). These core-shell type nanoparticles accumulate in tumors through the enhanced permeability and retention (EPR) effect and release L-Arg in macrophages, enabling controlled NO generation. This system achieves a biphasic anti-tumor effect: low concentrations promote angiogenesis, while high concentrations induce apoptosis [17,18].

We hypothesize that combining Nano^ARG^s with PD-1 Ab could synergistically enhance cancer immunotherapy by enhancing tumor accumulation at low concentrations and promoting TAM polarization and T cell activation at high concentrations. This biphasic strategy seeks to overcome the limitations of monotherapy and offers a novel and effective approach to cancer treatment.

In this study, we first explore the combination of Nano^ARG^s and PD-1 Ab for cancer immunotherapy. By targeting TAMs and leveraging the PD-1/PD-L1 axis, we seek to amplify anti-tumor immune responses and reshape the tumor microenvironment. This innovative approach holds promise for achieving enhanced therapeutic outcomes and paving the way for advancing clinical applications.

## 2. Materials and methods

### 2.1. Materials

The α-methoxy-ω-amino-poly(ethylene glycol) (MeO-PEG-NH_2_ with a molecular weight of 5,000) was prepared according to our previous articles [18].

N^δ^-Benzyloxycarbonyl-L-ornithine (L-Orn(Z)) was purchased from Watanabe Chemical Industries, Ltd. (Hiroshima, Japan). Bis(trichloromethyl) carbonate (triphosgene) was purchased from Tokyo Chemical Industry Co., Ltd. (Tokyo, Japan). Superdehydrated tetrahydrofuran (THF) and superdehydrated N,N-Dimethylformamide (DMF) were purchased from Kanto Chemical Co., Inc. (Tokyo, Japan). Hexane and (+)-α-pinene, N,N-diisopropylethylamine (DIPEA) and N-methylpyrrolidone (NMP) were purchased from Wako Pure Chemical Industries, Ltd. (Wako, Osaka, Japan). Trifluoroacetic acid (TFA), hydrogen bromide (HBr; 30% in acetic acid), and

N,N′-bis(tert-butoxycarbonyl)-1 H-pyrazole-1-carboxamidine (PCX(Boc_2_)) were all purchased from Tokyo Chemical Industry, Co., Ltd., (Tokyo, Japan). Chondroitin sulfate (CS) was purchased from Beijing Solarbio Science & Technology Co. (China). C26 murine colorectal carcinoma cells were purchased from Hunan Yuguo Biotechnology Co., Ltd. (China). PD-1 antibody (PD-1 Ab) was purchased from Solelybio Biotechnology, Co., Ltd. (China). BALB/c mice were purchased from Beijing Sibeifu Biotechnology Co., Ltd. (China). Nitric oxide (NO) content detection kits, rat anti-mouse CD86 antibody, rabbit anti-mouse MMR/CD206 antibody, rabbit anti-mouse PD-L1/CD274 antibody, Alexa Fluor 488-conjugated goat anti-rat IgG and Alexa Fluor 488-conjugated goat anti-rabbit IgG were purchased from Beijing Solarbio Science & Technology Co., Inc. (China). PE/Cy-7-conjugated anti-CD19, APC-conjugated anti-CD3, FITC-conjugated anti-CD4, PerCP-Cy5.5-conjugated anti-CD8, FITC-conjugated anti-CD68, APC-conjugated anti-CD86, and PE-conjugated anti-CD206 were purchased from Thermo Fisher Scientific Inc., U.S.A..

### 2.2. Preparation of PEG-b-P(L-Arg)-based Nano^ARG^s

PEG-b-P(L-Arg) was synthesized according to our previous report with a slight modification [17,18]. Briefly, a PEG-b-poly(L-ornithine) block copolymer was prepared by ring-opening polymerization of N^δ^-benzyloxycarbonyl-L-ornithine N-carboxyanhydride (NCA), followed by deprotection of the N^δ^-benzyloxycarbonyl group using HBr to obtain PEG-b-P(L-Orn). Subsequently, PEG-b-P(L-Arg) was synthesized by guanidinylation of the side-chain primary amino groups on the P(L-Orn) side chains of PEG-b-P(L-Orn) using N,N′-bis(tert-butoxycarbonyl)-1 H-pyrazole-1-carboxamidine. Notably, this procedure quantitatively converts amino groups to guanidium groups on the side chains of polypeptide segments under mild conditions. The product was purified by dialysis against water and finally freeze-dried to obtain white powder.

PEG-b-P(L-Arg)-based Nano^ARG^s were prepared following these steps. First, the HCl salt of PEG-b-P(L-Arg) was dissolved in 10 mM HEPES – NaOH buffer (pH 7.4) to make a PEG-b-polycation stock solution. Chondroitin sulfate (CS) was dissolved separately in the same buffer to prepare a polyanion stock solution. The two solutions were mixed at a cation/anion (C/A) ratio of 1.0, where the C/A ratio was defined as the molar ratio of guanidino groups (C) on the PEG-b-P(L-Arg) side chains to the carboxylic and sulfonic groups (A) on the polyanion. The mixture was then vigorously vortexed and incubated at room temperature for at least 30 min to ensure the formation of the Nano^ARG^s. The hydrodynamic diameter of Nano^ARG^ was determined by dynamic light scattering (DLS) using a Zetasizer Nano Z, (Malvern Instruments Ltd., Worcestershire, UK).

### 2.3. Experimental protocol for dynamic CCK-8 monitoring of biphasic effect of Nano^ARG^s on proliferation of C26 colon cancer cells

To verify the effect of Nano^ARG^s on the proliferation of C26 colon cancer cells, C26 cells in the logarithmic growth phase with approximately 70% confluency were first trypsinized, collected by centrifugation, and adjusted to a density of 1 × 10^4^ cells/well for seeding in 96-well plates. The plates were incubated at 37°C with 5% CO₂ for 24 h to allow cell adhesion. Subsequently, the old medium was discarded, and fresh medium containing Nano^ARG^s at concentrations of 0, 10, 50, and 100 μM was added. A blank control group (cells treated with complete medium without Nano^ARG^s) and low-, medium-, and high-dose groups (cells treated with 10, 50, and 100 μM Nano^ARG^s) were established, with 5–6 replicate wells per group. The cultures were continued, and CCK-8 assays were performed at 0, 24, 48, and 72 h. Specifically, 10 μL of CCK-8 reagent was added to each well, followed by incubation at 37°C in the dark for 1–2 h. The absorbance at 450 nm was then measured using a microplate reader. A proliferation curve was generated with time as the x-axis and OD values as the y-axis to evaluate the biphasic effects of different Nano^ARG^s concentrations on cell proliferation.

### 2.4. Evaluation of cellular uptake of Nano^ARG^s in cancer cells

To investigate the cellular uptake of Nano^ARG^ in cancer cells, C26 colon cancer cells were first digested and seeded into 6-well plates or confocal dishes at a density of 1 × 10^5^ cells/well, then incubated at 37°C with 5% CO₂ until reaching 60%-70% confluency. After washing with preheated PBS, cells were treated with complete medium containing FITC-labeled Nano^ARG^s (100 μg/mL), and incubated for 2 h, 4 h, and 6 h, respectively. The reaction was terminated by washing with pre-cooled PBS, followed by fixation with 4% paraformaldehyde for 15 min and nuclear staining with DAPI for 5 min. After further washing, images were acquired using a confocal microscope at 488 nm (FITC) and 405 nm (DAPI) channels to observe the intracellular distribution of Nano^ARG^, or flow cytometry was used to quantitatively analyze the uptake efficiency by detecting fluorescence intensity, with a blank control group without Nano^ARG^ set simultaneously.

### 2.5. In vivo anti-tumor activity studies

Tumor-bearing mice were obtained by subcutaneously injecting 100 μL of a suspension containing 1 × 10^5^ C26 murine colorectal carcinoma cells to the right thigh of BALB/c mice. Tumors were allowed to grow until they reached a volume of approximately 100 mm^3^ (about seven days post-inoculation). Mice were then anesthetized with sodium pentobarbital (40 mg/kg) before treatments were administered as follows:

Nano^ARG^s Administration:

Nano^ARG^s were administered intravenously at a dose of 16 mg/kg of L-arginine in the Nano^ARG^s. Groups of mice received different numbers of injections (1–4) at 24-h intervals:

1-injection group (Nano^ARG^(1)): Day 0 (total dose 16 mg/kg).

2-injection group (Nano^ARG^(2)): Days 0 and 1 (total dose 32 mg/kg).

3-injection group (Nano^ARG^(3)): Days 0, 1, and 2 (total dose 48 mg/kg).

4-injection group (Nano^ARG^(4)): Days 0, 1, 2, and 3 (total dose 64 mg/kg).

Combination Therapy: For evaluating the synergistic anti-tumor effects, mice received intravenous injections of Nano^ARG^s (total dose 64 mg/kg based on L-arginine) and intraperitoneal injections of PD-1 Ab (5 mg/kg per injection, 4 injections total) on days 0, 1, 2, and 3.

Evaluation Metrics:

The anti-tumor effects of Nano^ARG^s and the synergistic effects of Nano^ARG^s combined with PD-1 Ab were assessed by measuring tumor size and body weight. Tumor volume was calculated as an ellipsoid using the formula:

V = a × b^2^/2 where a and b are the major and minor axes of the tumor (in mm) measured with a caliper.

Body weight changes were recorded as an indicator of systemic cytotoxicity.

### 2.6. Measurement of NO levels in serum and tumor tissues

Anesthetize tumor-bearing mice to facilitate the blood collection. Collect blood via cardiac puncture into anticoagulant-treated centrifuge tubes to prevent clotting. Centrifuge the samples at 3000–5000 rpm for 10–15 min to separate the plasma from cells. Carefully extract the supernatant plasma and store it at −80°C. First, prepare the NO content detection kit reagents and standards according to the manufacturer’s instructions. Subsequently, pipette plasma samples and standards into the designated wells of a microplate. Incubate the microplate under the specified conditions, then add the detection reagent. Determine plasma NO concentration based on the standard curve. Handle all samples carefully and adhere to the kit protocol to ensure reliable results.

Carefully dissect and extract the tumor tissues, removing surrounding non-tumor tissues and blood. Weigh the tissues and rinse them with ice-cold PBS. Homogenize the tumor tissues on ice to obtain a homogenate, then centrifuge at an appropriate speed and duration to collect the supernatant. NO levels in tumor tissue supernatants were measured using the same protocol as plasma NO detection.

### 2.7. Immunofluorescence

To evaluate the role of TAMs in tumor immunity and the immunomodulatory effect of PD-1 Ab, macrophage infiltration and PD-L1 expression were assessed using immunofluorescence. Tumor paraffin sections were deparaffinized and stained with the following primary antibodies: rat anti-mouse CD86 antibody (1:500) and rabbit anti-mouse MMR/CD206 (1:100) to identify TAMs, and rabbit anti-mouse PD-L1/CD274 antibody to evaluate PD-L1 expression. Secondary staining was performed with Alexa Fluor 488-conjugated goat anti-rat IgG (1:500) and Alexa Fluor 488-conjugated goat anti-rabbit IgG (1:500). All steps were performed following standard protocols, ensuring accurate visualization of TAM infiltration and PD-L1 levels in tumor sections.

### 2.8. Flow cytometry

BALB/c mice were inoculated with 1 × 10^5^ C26 cells. When tumor volume reached approximately 100 mm^3^ post-inoculation, tumor tissues were harvested (*n* = 5). The tissues were prepared for flow cytometry by digestion with a tumor dissociation kit (MACS) and disrupted into single-cell suspensions using a 70 μm cell strainer. The resulting cells were washed, pelleted, and resuspended in D-PBS. For flow cytometric analysis, 1 × 10^6^ cells from each sample were incubated with the following antibodies: PE/Cy-7-conjugated anti-CD19, APC-conjugated anti-CD3, FITC-conjugated anti-CD4, PerCP-Cy5.5-conjugated anti-CD8, FITC-conjugated anti-CD68, APC-conjugated anti-CD86, PE-conjugated anti-CD206.

### 2.9. Statistical analysis

All values were expressed as the mean ± standard error of the mean (SEM). Student’s t-test was used to compare groups and evaluate statistical differences between means. *p* < 0.05 was considered statistically significant.

## 3. Results and discussion

### 3.1. Characteristic of PEG-b-P(L-Arg)-based Nano^ARG^s

**Figure 1.**
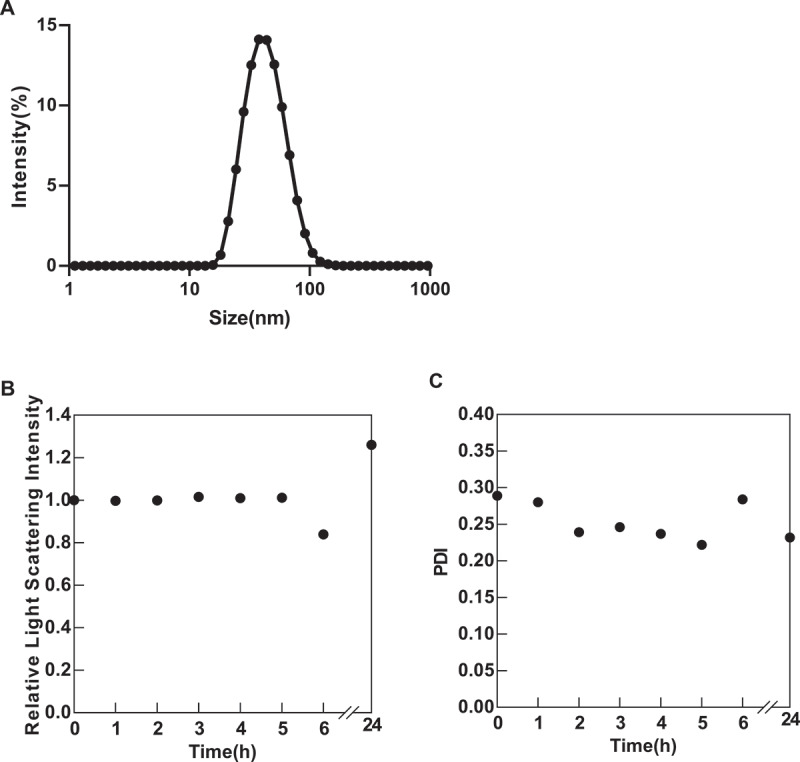
A, size of Nano^ARG^ determined by DLS; B-C,Relative LSI and PDI of Nano^ARG^in NaCl at a concentration of 150 mM against 24 h, respectively.

To investigate the physical properties and stability of the newly synthesized Nano^ARG^s, we first characterized their fundamental characteristics using various analytical techniques. Dynamic Light Scattering (DLS) was used to determine the hydrodynamic size and zeta potential at 25°C. The nanoparticles showed a hydrodynamic size of approximately 43.2 ± 1.3 nm and a zeta potential of −9.07 ± 0.26 mV (Table 1), as presented in the size-intensity distribution curve (Figure 1A). Given that ionic strength can significantly influence the stability of polyion complexes, we evaluated the colloidal stability of Nano^ARG^s in a PBS buffer containing 150 mM NaCl. After 24 h of incubation, over 90% of the relative initial light scattering intensity (LSI) was retained (Figure 1B), indicating that the nanoparticles maintain high colloidal stability under physiological salt conditions. These findings provide essential information regarding the formulation quality and suitability of Nano^ARG^s for biological applications.

**Table 1.** Particle size, polydispersity index and zeta potential of Nano^ARG^ (*n* = 3).

Samples	Size (nm)	PDI	Zeta potential (mV)
Nano^ARG^	43.2 ± 1.3	0.16 ± 0.03	−9.07 ± 0.26

### 3.2. In vitro anti-tumor efficacy of Nano^ARG^s combined with PD-1 Ab in cellular experiments

**Figure 2.**
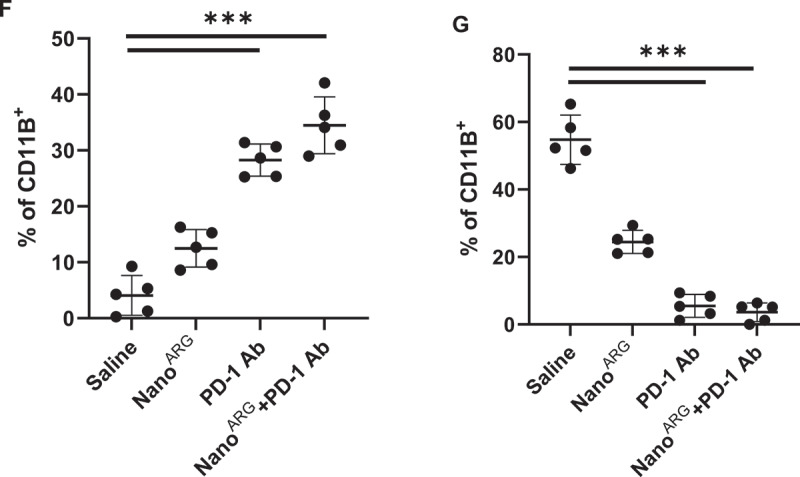
A, TUNEL assay results diagram illustration with different dose of Nano^ARG^. Blue (DAPI): total cellnuclei; Green (TUNEL): apoptotic cells; merge overlap (Cyan/Teal): directly identifies apoptotic nuclei. Scale bar, 500 μm. B, fluorescence level was determined by measuring the mean green values of TUNEL signal using the image J software. C, C26 tumor cell viability assay results with different dose ofNano^ARG^. D-E, NO and IFN-γ levels in the system after co-culturing T cells in the upper chamber with a mixture of C26 cells and J774 macrophages in the lower chamber of the Transwell co-culture system, respectively. F-G, quantification of (F)M1 and (G) M2 macrophage polarization. Data are expressed as average ± SEM. Statistical significance is indicated as follows: ***p < 0.001, ns: not significant.

**Figure 3.**
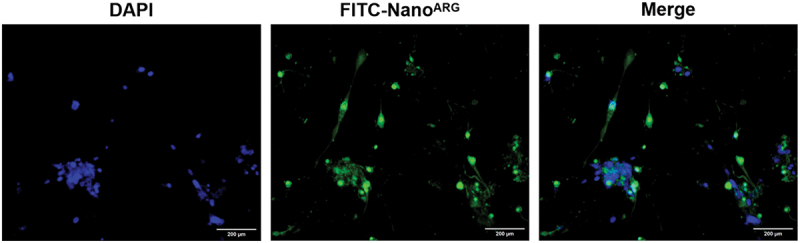
Fluorescence microscopy images illustrating the cellular uptake of FITC-labeled Nano^ARG^ in cancer cells. Nuclei were stained with DAPI (blue). Green fluorescence corresponds to FITC-Nano^ARG^, and merged images (merge) show the colocalization of Nano^ARG^ within cancer cells. Scale bar, 200 μm.

To establish a logical connection with the preceding discussion on synergistic mechanisms, we first characterized the dose-dependent effects of Nano^ARG^s on the proliferation of tumor cells *in vitro*, which serve as a foundation for exploring its combinatorial activity with PD-1 Ab. The TUNEL results (Figure 2A,b) showed that, compared with 10 μM, 50 μM and 100 μM Nano^ARG^s significantly increased the apoptosis rate of tumor cells. This might be because Nano^ARG^s generate different concentrations of NO, which in turn exerts distinct apoptosis-inducing effects on tumor cells. Meanwhile, the CCK8 cell experiment indicated that the survival rate of tumor cells decreased to approximately 50% (Figure 2C), revalidating the dose-dependent effect of NO levels on tumor cell proliferation. These findings are consistent with the results of animal experiments, where low-dose Nano^ARG^s promoted tumor growth [19,20], while high-dose Nano^ARG^s inhibited tumor growth [21,22]. We also verified the cellular uptake of Nano^ARG^s in tumor cells using fluorescence labeling. As shown in Figure 3, cancer cells nuclei were stained with DAPI (blue), and Nano^ARG^s were labeled with FITC (green). The merged images clearly demonstrated the internalization of FITC-Nano^ARG^s by tumor cells, as evidenced by the colocalization of green (nanoparticles) and blue (cell nuclei) signals. Collectively, the dose-dependent effects of Nano^ARG^s on tumor cell proliferation and apoptosis, along with confirmed cellular uptake in tumor cells lays a solid foundation for further exploring its combined application with PD-1 Ab in anti-tumor therapy.

In the exploration of the synergistic mechanism between Nano^ARG^s and PD-1 Ab, our hypothesis is as follows: Arginine, a key substrate for NO synthesis, can be sufficiently supplied by Nano^ARG^s within M1 macrophages. This process enables inducible iNOS in the cells to catalyze arginine, generating excessive NO, which subsequently kills tumor cells [3]. The underlying mechanism is that high-concentration NO specifically deaminates guanine on DNA into xanthine through a deamination reaction. This base modification can cause DNA structural damage, mediate genotoxicity, and interfere with the normal proliferation and repair processes of tumor cells [22]. On the other hand, PD-1 Ab blocks the PD-1/PD-L1 immune checkpoint signaling pathway, relieving the immunosuppression of tumor cells on T cells and activating the anti-tumor activity of T cells [23–27]. Activated T cells secrete cytokines such as IFN-γ [28,29], among which IFN-γ can induce a phenotypic switch in TAMs, promoting the transformation of immunosuppressive M2 macrophages into anti-tumor M1 macrophages [30–32]. M1 macrophages not only directly phagocytose and kill tumor cells but also enhance the immune response by secreting cytotoxic substances [33,34]. More importantly, the increase in the number of M1 macrophages provides a more favorable cellular environment for NO production [3], thus forming a positive feedback loop of ‘increased M1 macrophages-enhanced NO production-augmented tumor killing’.

Based on the above theory, we designed an *in vitro* co-culture experiment. C26 tumor cells, T cells, and J774 macrophages were co-cultured, and experimental groups treated with Nano^ARG^s, PD-1 Ab, and their combination were set up.

Based on the experimental results of Figure 2D, the NO level in the Nano^ARG^s group was significantly higher than that in the control group, confirming that Nano^ARG^s can effectively promote NO production by M1 macrophages. Although the NO level in the PD-1 Ab group increased, it was lower than that in the Nano^ARG^s group. Notably, the combined use of Nano^ARG^s and PD-1 Ab led to a further significant increase in NO levels, indicating that after PD-1 Ab induces an increase in M1 macrophages, it synergizes with Nano^ARG^s to enhance NO production. In the detection of IFN-γ levels (Figure 2E), the PD-1 Ab group showed significantly higher levels than the control group, consistent with the mechanism of PD-1 Ab activating T cells to secrete IFN-γ. There was no significant difference in IFN-γ levels between the Nano^ARG^ group and the control group, suggesting that Nano^ARG^ has limited effect on T cell secretion of IFN-γ. The IFN-γ level in the combined group was higher than that in the PD-1 Ab monotherapy group, possibly due to the synergistic effect of NO and M1 macrophages in enhancing T cell activation, amplifying IFN-γ secretion, and thereby promoting the M2-to-M1 phenotypic transition.

**Figure:**
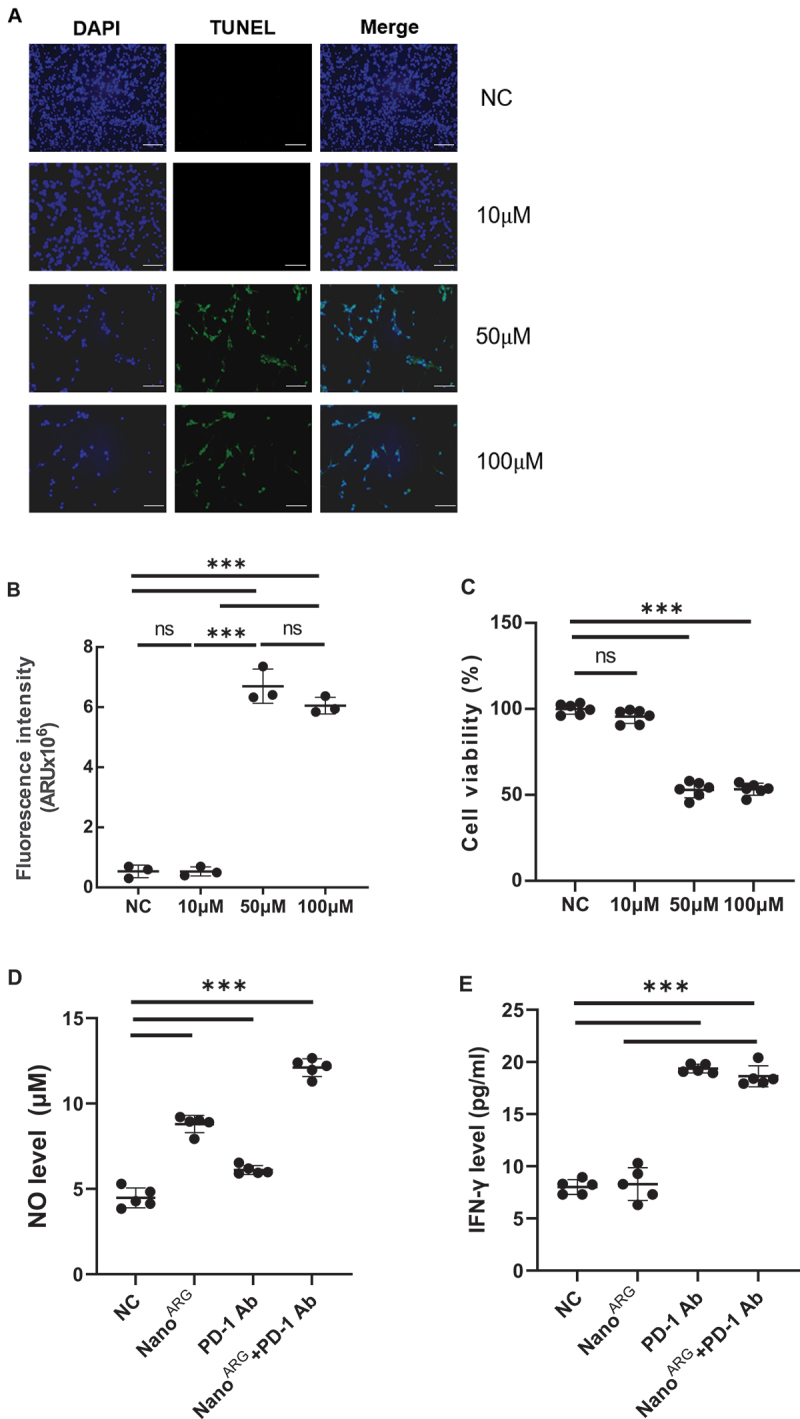

To further verify that the PD-1 Ab exerts its immunomodulatory effects by blocking the PD-1/PD-L1 checkpoint signaling pathway, relieving tumor-mediated immune suppression of T cells, and activating T cells to secrete IFN-γ for M2 to antitumor M1 conversion, we evaluated the polarization status of macrophages within a co-culture cell system. As presented in Figure 2F,G, which illustrates the phenotypic profiling of macrophage within the tumor microenvironment across distinct treatment regimens, via flow cytometry-based quantification of M1-type (CD86^+^) and M2-type (CD206^+^) subpopulations. In the saline treated control group, macrophages exhibited a predominant M2-polarized phenotype, indicative of a protumor immunosuppressive microenvironment. Treatment with Nano^ARG^ induced a modest shift toward M1 polarization (increased CD86^+^ cells), yet retained a substantial M2 type population. Administration of PD-1 Ab alone promoted a partial transition to M1 phenotype, as evidenced by elevated CD86^+^ frequencies relative to saline and Nano^ARG^ groups. Notably, the combination of Nano^ARG^ and PD-1 Ab elicited a profound reprogramming of macrophages: a marked enrichment of M1 subpopulations (manifested by a cluster of data points at higher CD11B^+^ proportions) and a near-complete depletion of

M2-type cells. Statistically significant differences highlight the robust immunomodulatory synergy of this combinatorial strategy, which remodels the myeloid cell landscape from a protumor to an antitumor state. This phenotypic shift aligns with and complements prior observations of dose-dependent tumor cell apoptosis (e.g. induced by Nano^ARG^-mediated NO signaling) and *in vivo* antitumor effects (low-dose promotion vs. high-dose inhibition), collectively underscoring a multi-modal mechanism of action wherein immunotherapy-nanotherapy combinations potentiate antitumor immunity via both direct apoptosis induction and tumor microenvironment reprogramming.

Overall, these results are highly consistent with the hypothesis that ‘PD-1 Ab activates T cells and induces macrophage phenotypic transition, while Nano^ARG^ supplies arginine to M1 macrophages to promote NO production, and the two form a positive feedback loop through synergy’, providing *in vitro* experimental evidence for the anti-tumor mechanism of the combined therapy with Nano^ARG^ and PD-1 Ab.

### 3.3. Anti-tumor effect of PEG-b-P(L-Arg)-based Nano^ARG^s

NO is widely recognized for its diverse, concentration-dependent functions in cancer therapy. At low concentrations, NO promotes angiogenesis by stabilizing hypoxia-inducible factor (HIF), activating pro-survival pathways (Akt and MAPK/ERK1/2), and upregulating pro-angiogenic factors [35]. Conversely, high concentrations of NO induce DNA damage, activate p53, and trigger apoptosis in tumor cells, underscoring its dual role in tumor progression and suppression [36].

**Figure 4.**
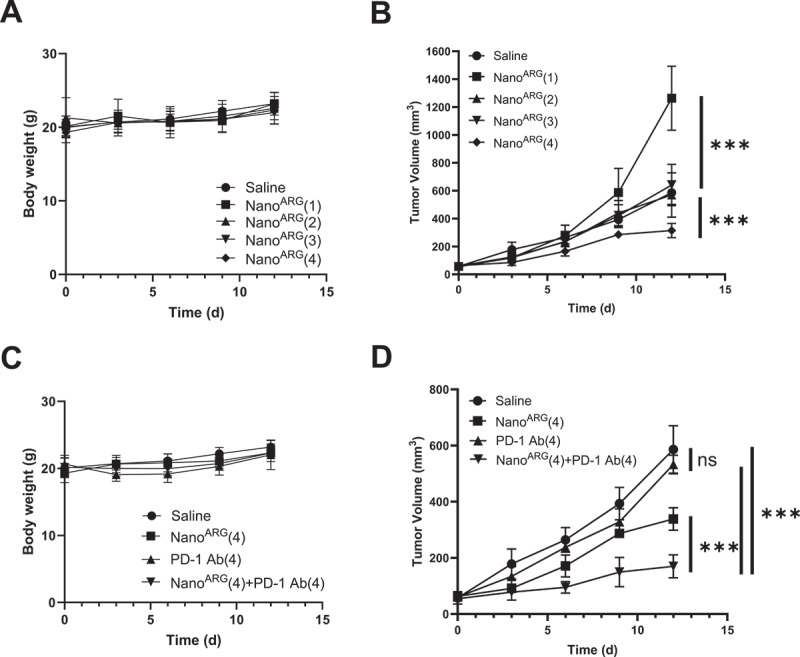
Average body weight (**A**) and tumor growth (**B**) in C26 murine colorectal carcinoma model following administration of: saline (control), Nano^ARG^(1) total dose of 16 mg/kg L-arginine, Nano^ARG^(2): total dose of 32 mg/kg L-arginine, (3) Nano^ARG^(3): total dose of 48 mg/kg L-arginine, Nano^ARG^(4): total dose of 64 mg/kg L-arginine.Average body weight (**C**) and tumor growth (**D**) in the same model upon administration of: saline (control).

In this study, we investigated the biphasic antitumor effects of Nano^ARG^s in C26 tumor-bearing mice, focusing on their ability to generate cytotoxic NO via iNOS in M1-type macrophages, similar to our previous study [18]. Tumor growth was monitored over 12 days following Nano^ARG^s administration (Figure 4). Mice treated with four intravenous injections of the Nano^ARG^s exhibited no significant weight loss (Figure 4A), indicating minimal systemic toxicity. Tumor size, however, varied significantly across dosing regimens (Figure 4B).

Low doses of Nano^ARG^ (a single injection at 16 mg/kg; Nano^ARG^ (1)) promoted accelerated tumor growth, likely due to NO’s pro-angiogenic effects at subtherapeutic concentrations. Intermediate doses (two 16 mg/kg injections; Nano^ARG^ (2)) and three injections (Nano^ARG^ (3)) resulted in tumor growth rates comparable to the control. In contrast, high dose (Nano^ARG^ (4)) effectively suppressed tumor growth.

These results underscore the critical importance of dose optimization. While low concentrations of NO may inadvertently facilitate tumor progression, higher doses can effectively suppress tumor growth. This study validates the potential of Nano^ARG^s as a biphasic anti-tumor therapy and highlights the need for precise dosing strategies to maximize therapeutic efficacy while minimizing adverse effects.

### 3.4. Synergistic anti-tumor effect of PEG-b-P(L-Arg)-based Nano^ARG^s and PD-1 Ab

Building on the results above, we investigated the *in vivo* antitumor effects of combination therapy using Nano^ARG^s and PD-1 Ab. In the C26 colon cancer model, Nano^ARG^(4) alone significantly inhibited tumor growth (*p* < 0.001). This inhibitory effect was further enhanced when combined with PD-1 Ab (*p* < 0.001), whereas PD-1 Ab monotherapy showed no significant effect on tumor growth (Figure 4D). The combination treatment exhibited minimal adverse effects, with no significant weight loss observed (Figure 4C), indicating a favorable safety profile.

The synergistic effect may stem from Nano^ARG^-induced NO production, which promotes immune cell infiltration and tumor-associated macrophage reprogramming, combined with PD-1 Ab-mediated immune checkpoint blockade. These complementary mechanisms likely amplify the anti-tumor response beyond what either therapy achieves individually. These findings underscore the potential of Nano^ARG^s and PD-1 Ab as a combination therapy, offering a promising strategy to overcome the limitations of monotherapy in cancer immunotherapy.

### 3.5. Increased NO levels in serum and tumor tissues induced by Nano^ARG^s

In tumor tissues, iNOS utilizes L-Arg and reactive oxygen species (ROS) as substrates to efficiently produce NO [37]. This process exerts a dual impact on tumor biology: at low concentrations, NO promotes angiogenesis and inhibits programmed cell death, facilitating tumor progression [19,20], while at high concentrations, NO induces DNA damage, p53 activation, and apoptosis in tumor cells [21,22]. This duality highlights the critical importance of fine-tuning NO production and distribution in designing effective anti-tumor therapies.

The Nano^ARG^ system, an L-Arg-based NO donor platform developed in this study, overcome challenges associated with NO’s physiological instability and delivery limitations [38]. We evaluated NO levels in serum and tumor tissues across different treatment groups, including Nano^ARG^s at varying doses, PD-1 Ab alone, and the combination therapy of Nano^ARG^s + PD-1 Ab.

**Figure 5.**
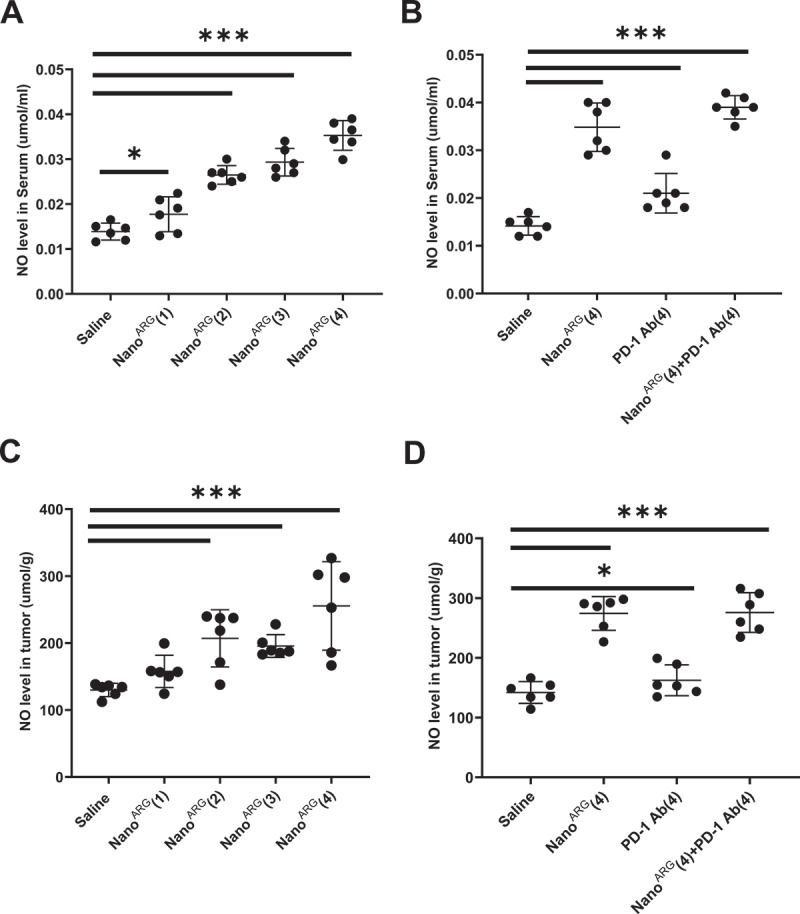
NO levels in serum (A, B) and tumor tissues (C, D) following administration of Nano^ARG^(4), PD-1 Ab(4), Nano^ARG^(4) + PD-1 Ab(4). Data are presented as mean ± SEM, (*n* = 6 mice per group). Statistical significance is indicated as follows: *p < 0.05, **p < 0.01, ***p < 0.001.

As shown in Figure 5A,C NO levels in serum and tumor tissues increased proportionally with Nano^ARG^ dose, confirming the effectiveness as a targeted NO donor. Interestingly, PD-1 Ab alone also elevated NO levels (Figure 5B,C), likely due to increased macrophage infiltration and enhanced arginine utilization [5]. This observation aligns with findings by Chiku et al., who reported that blocking the PD-1 pathway restored CD3^+^ T cell activity, increased iNOS expression, and elevated NO production in macrophages [39]. The combined treatment further amplified NO levels in serum and tumor tissues, providing a mechanistic explanation for its enhanced anti-tumor efficacy. These results underscore the importance of NO modulation in the combination therapies, offering a clear mechanistic basis for their potential clinical application.

### 3.6 NO promotes deep infiltration of immune cells in tumor tissues

**Figure 6.**
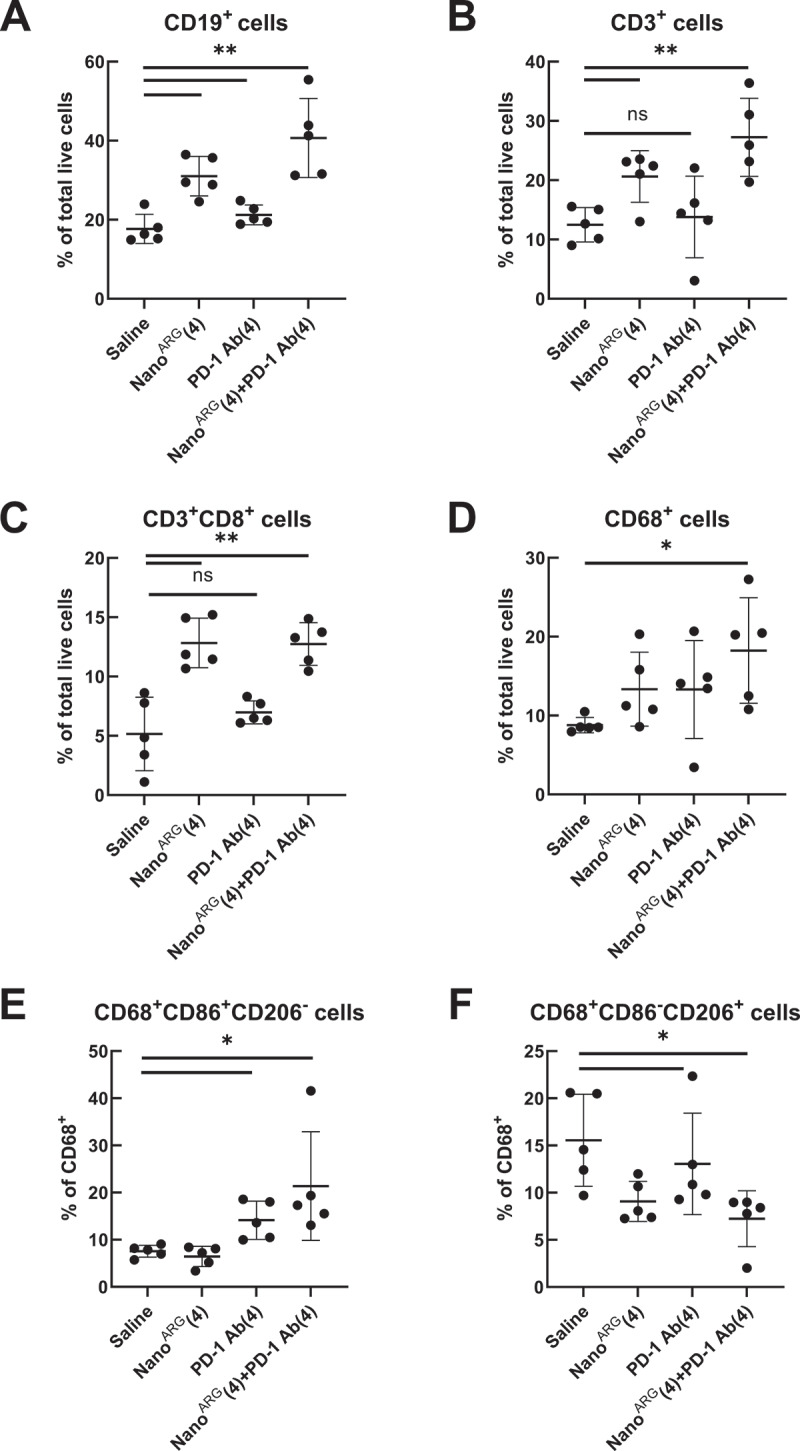
Quantification of immune cell populations in tumor tissues. **(A)** B cells (CD19^+^), **(B)** T cells (CD3^+^), **(C)** CD8^+^ T cells (CD3^+^CD8^+^), **(D)** Macrophages cells (CD68^+^), **(E)** M1 cells (CD68^+^CD86^+^CD206^−^), **(F)** M2 cells (CD68^+^CD86^−^CD206^+^). Data are presented as mean ± SEM (*n* = 5 per group), statistical significance is indicated as follows: *p < 0.05, **p < 0.01, ***p < 0.001, ns: not significant.

iNOS utilizes L-Arg as a substrate to produce cytotoxic NO, which normalizes tumor vasculature and facilitates deeper immune cell infiltration [40]. To evaluate the composition of tumor-infiltrating leukocytes, baseline tumor tissues from tumor-bearing mice were dissociated into single cell suspension and analyzed via flow cytometry (Figure 6). Treatment with Nano^ARG^s significantly increased immune infiltration, as evidenced by elevated levels of CD19^+^ B cells (Figure 6A) and CD3^+^CD8^+^ T cells (CD3^+^CD8^+^) (Figure 6B,C). Notably, NO produced by Nano^ARG^s enhanced T cell infiltration and cytotoxic activity, improving tumor-killing efficiency.

The infiltrated T cells provided additional targets for PD-1 Ab therapy. By binding to PD-1 on T cell surfaces, PD-1 Ab relieved immune suppression, restored signaling pathway activation, and reactivated T cell’s tumor-killing functions. These complementary mechanisms highlight the synergistic potential of combining Nano^ARG^s with PD-1 Ab in promoting deep immune infiltration and enhancing anti-tumor efficacy.

### 3.7 PD-1 Ab induced polarization of TAMs toward the M1 phenotype

The interaction between PD-1 and its ligand PD-L1 negatively regulates the immune response by inhibiting the activation, proliferation, and cytokine secretion of tumor antigen-specific T cells expressing PD-1. This interaction also induces T cell apoptosis, enabling immune evasion and tumor progression [41,42]. Blocking the PD-1/PD-L1 interaction with an antibody relieves immune suppression, restores T cell functionality, and generates effective anti-tumor response [23–27].

**Figure 7.**
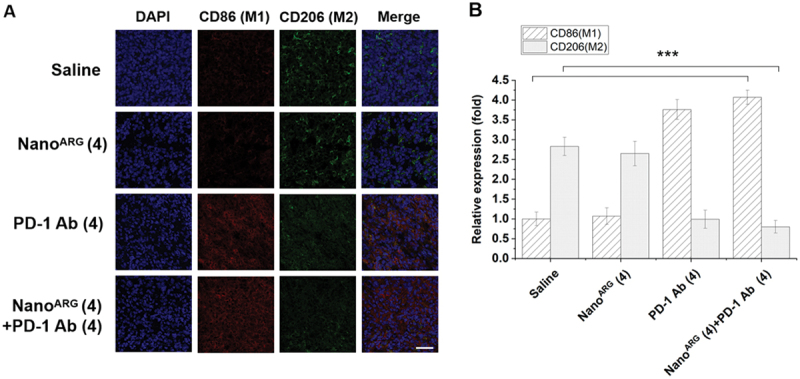
**A**, immunofluorescence staining of CD86 (M1) and CD206 (M2) in tumor tissues with Nano^ARG^ and PD-1 Ab. Scale bar, 50 µm. **B**, quantification of CD86 (M1) and CD206 (M2) using the image J software (*n* = 5 fields per tumor sample). Data are presented as mean ± SEM. Statistical significance is indicated as follows: ***p < 0.001.

Building on our findings related to NO, we investigated TAM polarization following PD-1 Ab treatment. TAMs, which are a major source of PD-L1 expression in the tumor microenvironment [43–45], often express higher PD-1 levels than tumor cells [46]. Our results showed that PD-1 Ab treatment significantly increased M1-type macrophages while reducing M2-type macrophages (Figures 6D,E,F and 7). This polarization shift aligns with the findings of Fu et al. [7], who demonstrated that combining Foretinib, a multi-receptor tyrosine kinase inhibitor, with an anti-PD-1 antibody enhanced anti-tumor immunity by increasing T cell infiltration, reducing TAMs, and inhibiting M2 polarization via the JAK2-STAT1 pathway.

These results highlight the potential of PD-1 Ab to mediate TAM phenotypic transformation, contributing to its anti-tumor effects by promoting M1 polarization and reshaping the tumor microenvironment. This dual role underscores the promise of PD-1 Ab as both a checkpoint inhibitor and an immune microenvironment modulator, paving the way for more effective combination therapies in cancer treatment.

### 3.8. PD-1 Ab reactivates T cells to exert anti-tumor effects

In the tumor microenvironment, tumor cells express large quantities of PD-L1. When PD-L1 on tumor cells binds to PD-1 on tumor-infiltrating T cells, it effectively shields the tumor cells from immune recognition, suppressing anti-tumor activity of T cells, and facilitating immune escape [47]. This mechanism plays a critical role in tumor immune evasion. PD-1 Ab disrupts this interaction, reactivating suppressed T cells and restoring their anti-tumor functions [48].

**Figure 8.**
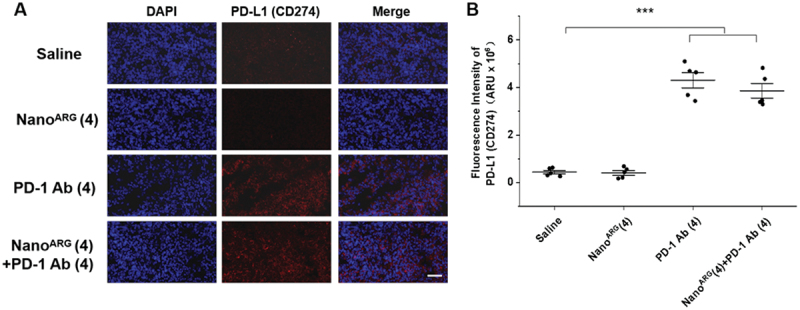
**A**. immunofluorescence staining of PD-L1 (CD274) in tumor tissues treated with of Nano^ARG^, PD-1 Ab, and Nano^ARG^(4)+PD-1 Ab(4). Scale bar, 50 μm. **B**, quantification of CD86 (M1) and CD206 (M2) using the image J software, (*n* = 5 fields per tumor sample). Data are presented as mean ± SEM. Statistical significance is indicated as follows: *p < 0.05, **p < 0.01..

To evaluate the effect of PD-1 Ab, we assessed PD-L1 expression on tumor cell surfaces. Both PD-1 Ab alone and its combination with Nano^ARG^s significantly increased PD-L1 expression (Figure 8), likely reflecting heightened immune activation. This increase was accompanied by enhanced T cell infiltration and cytotoxic activity in the tumor microenvironment, resulting in more effective cancer cell elimination [49].

By binding PD-1 on T cells, PD-1 Ab relieves immune suppression and reactivates key signaling pathways essential for T cell function [50]. Reactivated T cells regain their ability to:

- Recognize tumor antigens and initiate an immune response [51].
- Secretion of perforin and granzyme to damage target cell membranes [52].
- Activation of other immune cells through cytokine release, such as INF-γ, to coordinate immune defense and clearance [29,53,54].

In summary, PD-1 Ab restores the immune function of T cells, enabling them to exert robust cytotoxic and immune-modulatory effects. When combined with Nano^ARG^, these effects are further amplified, providing a potent strategy to overcome immune evasion and enhance anti-tumor immunity.

### 3.9 Systemic administration of Nano^ARG^s and PD-1 Ab shows no toxicity in mice

**Figure 9.**
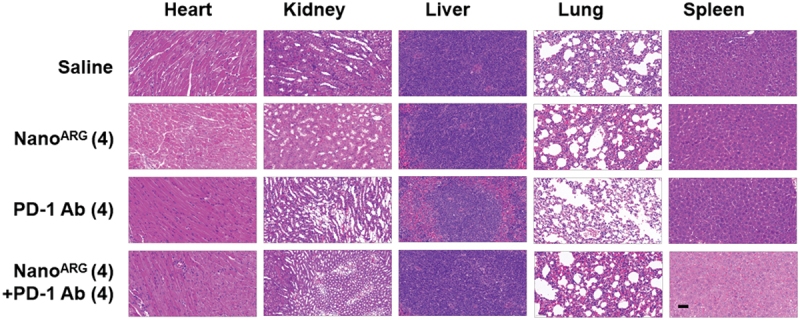
H&E staining of organs from C26 tumor-bearing mice treated with PD-1Ab(4), Nano^ARG^(4), Nano^ARG^(4) + PD-1 Ab(4) or saline. Scale bar, 50 μm.

The potential toxicity of the therapy was evaluated through histological analysis of H&E-stained tissues sections from major organs, including the liver, kidney, heart, lung, and spleen. In the C26 murine colorectal carcinoma model, microscopic examination revealed no signs of organ damage, including thrombosis or hemorrhage, in mice treated with Nano^ARG^(4), PD-1 Ab(4), or their combination (Nano^ARG^(4) + PD-1 Ab(4)), compared to the control group (Figure 9). All tissues scored 0 on a histological grading scale for inflammation, necrosis, and thrombosis, indicating the absence of detectable damage. These findings demonstrate that systemic administration of Nano^ARG^(4), PD-1 Ab(4) or their combination is well tolerated in mice, with no detectable toxicity under the tested conditions. While these results underscore the safety of the combination therapy, further studies are warranted to evaluate potential long-term or high-dose effects, supporting its potential for safe clinical translation. To our knowledge, this is the first report to experimentally demonstrate a synergistic feedback mechanism involving PD-1-induced macrophage polarization and NO-mediated cytotoxicity.

## 4. Conclusion and discussion

In this study, we have demonstrated the promising potential of combining PEG-b-P(L-Arg)-based Nano^ARG^s with PD-1 Ab as a novel cancer immunotherapy strategy. Our key findings underscore the multi-faceted advantages of this approach, integrating both a discussion of technological limitations and conclusions about research significance.

Traditional tumor-targeted nanocarriers such as liposomes and polymeric nanoparticles typically rely on passive accumulation via the EPR effect or active targeting through surface-conjugated ligands like antibodies, but these systems are plagued by inherent limitations. These include complex synthesis processes, poor tumor penetration due to suboptimal size (particles smaller than 20 nm are rapidly filtered by the kidneys, while those larger than 100 nm exhibit limited intratumoral diffusion), rapid clearance by the reticuloendothelial system, and uncontrolled drug release. In contrast, Nano^ARG^s overcome these challenges through rational design: its 43.2 ± 1.3 nm diameter with a slightly negative surface charge of −9.07 ± 0.26 mV enables efficient tumor accumulation via the EPR effect, while active uptake by tumor-associated J774 macrophages leverages endogenous tumor biology rather than synthetic targeting ligands. The iNOS-catalyzed in situ generation of NO eliminates the need for traditional drug loading and release mechanisms, offering unique advantages such as enhanced tumor targeting via macrophage-mediated uptake, controlled NO generation through enzyme catalysis, reduced systemic toxicity due to localized activation, and a simplified single-component formulation.

Our research has yielded several critical conclusions about the therapeutic mechanisms at play. First, Nano^ARG^s exhibit a biphasic antitumor effect: low doses tend to promote tumor growth through pro-angiogenic NO signaling, while high doses effectively inhibit tumor growth via DNA-damaging NO cytotoxicity, a phenomenon validated in both in vitro and in vivo models. Second, the combination of Nano^ARG^s and PD-1 Ab demonstrates synergistic anti-tumor activity, outperforming individual monotherapies by inducing the polarization of tumor-associated macrophages from the immunosuppressive M2 phenotype to the cytotoxic M1 phenotype. This process is accompanied by increased NO production and reactivation of T cells, creating a positive feedback loop of ‘M1 macrophage increase – NO elevation – enhanced tumor killing’. Notably, this combination therapy does not lead to significant adverse reactions and is well-tolerated by mice, highlighting its potential for clinical translation.

Overall, this study establishes Nano^ARG^s as a paradigm-shifting platform for integrating metabolic modulation with immunotherapy, providing innovative insights and strategies for cancer treatment by emphasizing the importance of modulating the tumor microenvironment and regulating the immune system. Looking to the future, ongoing research should focus on several key areas: elucidating the molecular mechanisms underlying NO-mediated tumor cell killing and immune activation, validating these findings in additional tumor models to enhance generalizability, and optimizing treatment regimens to balance NO release kinetics with immune checkpoint blockade timing. These efforts will be crucial for advancing Nano^ARG^-based combination therapies toward broader clinical applications and improving cancer treatment outcomes.
